# Thymic Function Associated With Cancer Development, Relapse, and Antitumor Immunity – A Mini-Review

**DOI:** 10.3389/fimmu.2020.00773

**Published:** 2020-04-30

**Authors:** Weikan Wang, Rachel Thomas, Olga Sizova, Dong-Ming Su

**Affiliations:** ^1^Cell Biology, Immunology, and Microbiology Graduate Program, Graduate School of Biomedical Sciences, University of North Texas Health Science Center, Fort Worth, TX, United States; ^2^Department of Hematopoietic Biology and Malignancy, The University of Texas MD Anderson Cancer Center, Houston, TX, United States; ^3^Department of Microbiology, Immunology, and Genetics, University of North Texas Health Science Center, Fort Worth, TX, United States

**Keywords:** thymic involution, negative selection and regulatory T (Treg) cell generation, cancer immunity, tumor microenvironment, tumor reservoir

## Abstract

The thymus is the central lymphoid organ for T cell development, a cradle of T cells, and for central tolerance establishment, an educator of T cells, maintaining homeostatic cellular immunity. T cell immunity is critical to control cancer occurrence, relapse, and antitumor immunity. Evidence on how aberrant thymic function influences cancer remains largely insufficient, however, there has been recent progress. For example, the involuted thymus results in reduced output of naïve T cells and a restricted T cell receptor (TCR) repertoire, inducing immunosenescence and potentially dampening immune surveillance of neoplasia. In addition, the involuted thymus relatively enhances regulatory T (Treg) cell generation. This coupled with age-related accumulation of Treg cells in the periphery, potentially provides a supportive microenvironment for tumors to escape T cell-mediated antitumor responses. Furthermore, acute thymic involution from chemotherapy can create a tumor reservoir, resulting from an inflammatory microenvironment in the thymus, which is suitable for disseminated tumor cells to hide, survive chemotherapy, and become dormant. This may eventually result in cancer metastatic relapse. On the other hand, if thymic involution is wisely taken advantage of, it may be potentially beneficial to antitumor immunity, since the involuted thymus increases output of self-reactive T cells, which may recognize certain tumor-associated self-antigens and enhance antitumor immunity, as demonstrated through depletion of autoimmune regulator (*AIRE*) gene in the thymus. Herein, we briefly review recent research progression regarding how altered thymic function modifies T cell immunity against tumors.

## Introduction

T cells are key players in cell-mediated antitumor immunity (1–4) as they have a diverse TCR repertoire specifically recognizing tremendous numbers of tumor neo-antigens, termed TSAs (5, 6), resulting from genomic mutations or viral infection. They can directly kill malignant cells in cytotoxic manners (1, 7, 8) and interact with other tumor-infiltrating immune cells (9) influencing immune surveillance. T cells are thymus-derived, heterogeneous lymphocytes, mainly including αβ-TCR CD4^+^/CD8^+^ and γδ-TCR T cells (10). As αβ-TCR T cells are the most abundant and comprehensively studied sub-population involved in antitumor immunity, we focus on this population.

The thymus mediates T cell development and the signals received by thymocytes from thymic stromal cells, primarily TECs, determine thymocyte fate. For example, Notch ligands expressed by TECs provide continuous Notch signals to thymocytes to decide each stage of T-lineage development (11, 12). Interleukin (IL)-7 is a second indispensable factor produced by TECs for the survival, proliferation and differentiation in early stages of T cell development (13, 14). After the completion of TCR rearrangement, the development and differentiation of T cells depend on the interaction between TCR and major histocompatibility complex (MHC)/self-antigens. This interaction leads to establishment of central tolerance via negative selection and regulatory T (Treg) cell selection (15–17). Thymic involution induced by primary TEC defects affects this signaling by impacting lymphostromal interactions. The process of T cell development in the thymus is complex, but there are several important checkpoints that decide successful establishment of immune surveillance and antitumor immunity: (a) αβ-TCR rearrangement to acquire various specificities of antigen recognition; (b) positive selection to achieve MHC restriction; and (c) negative selection/Treg cell generation to establish central tolerance to self (13, 17).

Thymic involution resulting from primary TEC defects occurs in the age-related phenotype, and not only reduces output of naïve T cells (18, 19), but also perturbs the interactions between MHC-II/self-peptide complexes on mTECs and TCRs on thymocytes, thereby altering TCR signaling strength, which impairs thymic negative selection and relatively enhances CD4^+^ thymic Treg (tTreg) cell generation (20, 21). These changes could lead to declined tumor immune surveillance, potentially attributed to a reduced capacity to recognize neo-antigens and deplete neoplasia. On the other hand, deliberately increasing release of self-reactive conventional T (Tcon) cells that are able to recognize tumor-borne self-antigens could enhance antitumor immunity (22–24). In addition, during aging, the involuted thymus generates relatively increased polyclonal tTreg cells (20), which, coupled with accumulated peripheral Treg (pTreg) cells (25, 26), may infiltrate to tumor mass and establish a microenvironment that suppresses both CD8^+^ and CD4^+^ T cell-mediated antitumor immunity, facilitating tumor cell survival (16, 27, 28). This could be related to the higher cancer incidence observed in the elderly (29).

Further, tumor-bearing individuals could be afflicted with cancer-related contributors of acute thymic involution, including (a) increased apoptosis of TECs and thymocytes (30–34) and obstruction of thymocyte maturation (32, 35, 36); and/or (b) chemotherapy-induced non-malignant thymic cellular apoptosis and senescence response (37–39). These will further disrupt antitumor immunity by disrupting T cell development and creating a tumor reservoir in the involuted thymus, allowing for tumor cell dormancy and eventually metastatic relapse (37, 38).

Therefore, thymic conditions impacting T cell immunity are critical issues underlying the high risk for late-life tumor development and the effectiveness (or lack thereof) of antitumor immunotherapy. Revealing the relationship between thymic conditions and T cell-mediated antitumor immunity may facilitate further studies in tumor immunology.

## Thymic Involution Is Associated With Declined T Cell-Mediated Immune Surveillance of Tumors

Tumor immune surveillance is an interaction between tumor development and antitumor immunity. The process of tumor immunoediting has three phases: elimination, equilibrium, and escape (40, 41). Elimination is an effective process of immune recognition via antigen-specific identification, and responsiveness to remove neoplasia. However, if T cells are senescent and/or tumor cells evolve into less targetable variants by genetic mutation or epigenetic modifications, the adaptive immune system might only restrain tumor growth, reaching a state of equilibrium. As this process continues it results in the selection of tumor cell variants that are resistant to antitumor response, ushering in the escape phase (40, 41).

**FIGURE 1:**
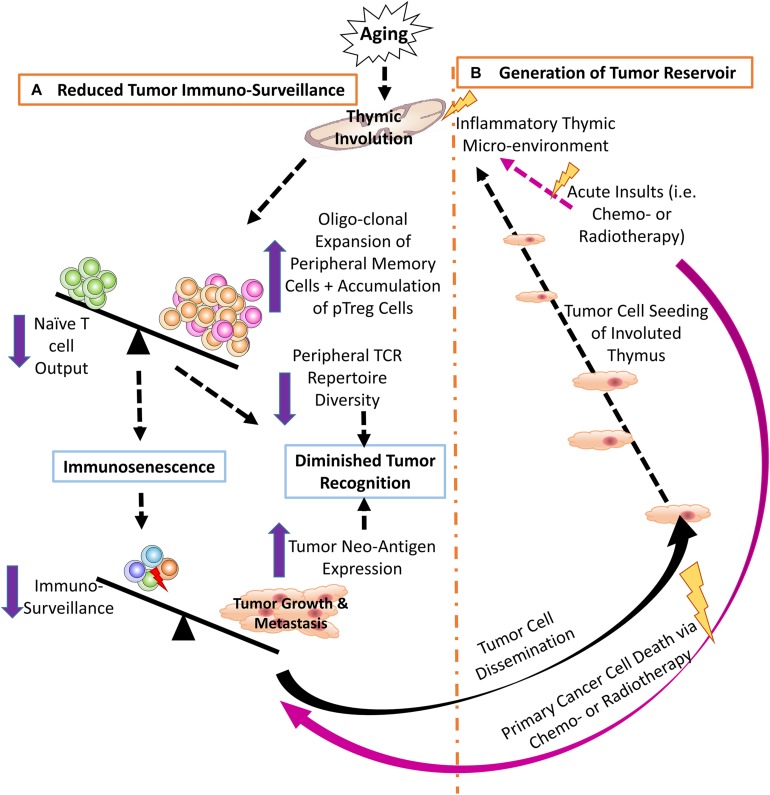
**(A)** Thymic involution contributes to reduced tumor immune surveillance by participating in immunosenescence and constricting the peripheral TCR repertoire diversity. Additionally, neo-antigens produced by either genomic mutation and/or viral infection create novel tumor antigens that may not be recognized by the reduced pool of naïve T cells in the aged periphery. **(B)** The involuted thymus acts as a pre-metastatic reservoir for disseminating tumor cells and the inflammatory thymic microenvironment promotes heterogeneous tumor cell dormancy, at both the cellular and population levels.

T cell immunosenescence is largely attributed to reduced output of naïve T cells from the aged, involuted thymus (18, 42–44), resulting in increased oligoclonal expansion of peripheral memory T cells (45, 46), thereby, restricting TCR repertoire diversity (47, 48). This hampers T cell ability to recognize tumor neo-antigens, resulting from high frequency of somatic mutations in proto-oncogenes and tumor suppressors in tumor cells, and/or from viral antigens produced by virus-induced cancers. These abnormal proteins are called TSAs (5), which are regarded by T cells as foreign antigens. Normally, the T effector (Teff) cell population can recognize tremendous numbers of tumor antigens (5, 6), while the senescent T cell population, with a reduced TCR repertoire diversity, might overlook these antigens. Therefore, one of the potential mechanisms of the reduced cancer immune surveillance is a compromised TCR repertoire generated first by the involuted thymus and exacerbated by age-related peripheral memory cell expansion, which neglects to recognize certain TSAs and fails to eliminate tumors (47, 49, 50) (Figure 1A).

Recent studies identified several senescent T cell markers: PD-1 and CD153 in murine senescence-associated T (SA-T) cells (51–53). Previously, our knowledge was limited to T cell secondary signaling molecule CD28, which is reduced or absent in senescent T cells. CD28^–neg^ “exhausted” peripheral T cells are accumulated in aged humans (54, 55). These T cells not only lose responsiveness to co-stimulation (56), but also are involved in chronic inflammation (57). The PD-1^+^CD153^+^ senescent T cells in mice also exhibit impaired TCR-mediated proliferation and defective IL-2 production, and are biased toward the secretion of pro-inflammatory cytokines, such as IFN-γ (45). It is not clear, however, whether increased PD-1 is directly involved in senescent T cell dysfunction. The generation of SA-T cells is generally attributed to thymic involution and the aged environment (53).

There are two major immunosuppressive mechanisms blocking antitumor immunity: the intrinsic PD-1—PD-L1 axis and the extrinsic Treg—Teff axis (58). A recent finding showed that senescent T cells express increased PD-1 (51–53, 59). This, coupled with increased PD-L1 on tumor cells (60), could lead to an enhanced PD-1/PD-L1 signaling (61), in which the interaction between PD-1/PD-L1 provides a possibility for the anergy, exhaustion, and apoptosis of tumor-reactive T cells (62), thereby, reducing cancer immune surveillance associated with senescent T cells (63). We will discuss the Treg—Teff axis in the following section.

Taken together, thymic involution, immunosenescence, and the declined TCR repertoire diversity, coupled with increased age-related genomic mutations in somatic cells and increased PD-1 expression on senescent T cells in the elderly, contribute to compromised immune surveillance of tumors and the higher late-life tumor incidence.

## Relatively Enhanced tTreg Cell Generation in the Involuted Thymus, Coupled With Accumulation of pTreg Cells, Potentially Suppress Antitumor Immunity

Thymic involution not only reduces naïve T cell output, but also relatively enhances tTreg generation as displayed by an increased ratio of tTreg versus tTcon in the aged, involuted thymus (20). The basic mechanism is potentially due to altered TCR signaling strength, which may skew CD4^+^ single positive thymocytes from negative selection to Treg cell generation in the involuted thymus (43). Strong TCR signaling strength, generated by interactions between MHC-II/self-peptide complexes and self-reactive TCRs, induce clonal depletion by negative selection, while intermediate TCR signaling strength induces thymocyte differentiation into CD4^+^FoxP3^+^ tTreg cells (17, 64, 65). MHC-II/self-peptide complexes expressed by mTECs are reduced due to mTEC decline in the involuted thymus, resulting in weakened interactions (20, 43, 66). Thus, some self-reactive T clones, which should be negatively selected with strong signaling, survive and differentiate into tTreg cells due to intermediate signaling (20). In addition, such skewing of thymocytes from clonal depletion to Treg generation could modify the TCR repertoire of Treg cells to include certain self-antigens that are also expressed by tumors, enabling these Treg cells to suppress antitumor immunity.

In light of the age-related accumulation of pTreg cells in the periphery (25) and the aging-related enhancement of FoxP3 expression (67), the underlying mechanisms may not be simply due to relatively enhanced tTreg cell generation in the involuted thymus, but also potentially due to declined activation of pro-apoptotic *BIM* gene (*Bcl2 homology-3, BH3-only*) (68) via increased methylation (68, 69) during aging. *BIM* should be activated after each immune reaction (after infection or inflammation, etc.) in order to deplete excess immune cells and return the expanded immune cell numbers to normal levels (70). However, with age, *BIM* activation in T cells is declined and homeostatic immune rebalance is hindered, resulting in an accumulation of “exhausted” senescent T cells and pTreg cells (25, 26, 71, 72). In addition, conversion of effector memory cells into memory Treg cells might occur in aged people (73). These all increase the pTreg pool (25, 74, 75).

Although Treg cells maintain immunological tolerance by suppressing excessive or aberrant immune responses mediated by Teff cells (76–78), they are opponents of antitumor immunity (79, 80) via their highly immunosuppressive functions against CD8^+^ cytotoxic T lymphocytes (CTLs) (27, 81, 82). Our current understanding is that Treg cells primarily infiltrate the tumor mass and execute suppressive function (77, 83, 84). Generally, T cell infiltration into the tumor mass correlates to tumor antigen expression. If the cancer mass expresses few neo-antigens, then greater numbers of Treg cells infiltrate to form a Treg-dominant tumor microenvironment; whereas, if the cancer mass expresses abundant neo-antigens, fewer Treg cells infiltrate, and more effector cells including CD8^+^ T cells can be primed and expand in the tumor tissues (16, 85, 86). Tumor-infiltrating Treg cells are thought to be recruited from the preexisting thymus-derived Treg population, including autoimmune regulator gene (*Aire*)-dependent TAA-specific Treg cells (87–89), rather than from peripherally induced tumor-specific Teff cells. Therefore, central tolerance is implicated in impaired antitumor responses.

Removal of Treg cells (with monoclonal antibodies, such as anti-CD25 (90), or other means) enhances T cell antitumor responses (15, 16, 91). However, anti-CD25 antibodies potentially eliminate activated Teff cells, expressing CD25 (92). Targeted functional inactivation of Treg cells based on constitutively expressed molecules including CTLA-4, GITR, TLR8 and OX40 (93–97) is a better means to nullify Treg cell function without decreasing Treg cell numbers from surrounding Teff cells (15), nor effecting Teff cell numbers. That is why anti-CTLA-4 (98) can serve as another immune checkpoint inhibitor to reduce Treg cell activation and be used for tumor immunotherapy (99).

Although direct evidence is still lacking about whether increased tTreg cells play a role in suppressing antitumor immunity, 80 – 95% of pTreg cells are derived from thymic generated tTreg cells bearing a thymic imprint (17, 64). Therefore, relative enhancement of tTreg cell generation resulting from thymic involution is a risk factor for suppressing antitumor immunity that ought not be overlooked.

## The Involuted Thymus Plays a Role as a Tumor Reservoir by Inducing Tumor Dormancy and Increasing the Risk for Eventual Metastatic Relapse

Metastatic relapse occurs when the same type of cancer recurs at a distant location (100) several years after removal of the primary tumor and adjuvant chemotherapy (101, 102), and this results mainly from chemo-resistance obtained by cancer cells in an inflammatory microenvironment (37, 38). Relapse, an immense clinical challenge, is responsible for 90% of cancer-associated mortality (103, 104). It means that cancer cells may still exist for a silent period after the primary treatment. Tumor pre-metastatic niches or reservoirs permit these silent cancer cells to hide and acquire chemo-resistance. Recently, several organs, such as the perivascular space of blood vessels in the lung and liver (105, 106) and bone marrow (107, 108), have been determined to be such reservoirs. We (37) and others (38) found that the involuted thymus is another tumor reservoir that allows for silent primary tumor cells to find safe-harbor.

Cancer cells circulating in the blood stream (109, 110) enter the thymus creating a heterogeneous environment, including malignant cancer cells and non-malignant thymic cells (TECs and thymocytes). Since the thymus contains mostly immature T cells and possesses semi-immune privilege, the cancer cells cannot be thoroughly eradicated by immune surveillance (37). In addition, the thymus is sensitive to many insults that cause involution. One of strongest insults is chemotherapy. In addition to killing cancer cells, systemic chemotherapy also results in non-malignant cell death and/or senescence due to DNA damage (111, 112), which produces an inflammatory microenvironment. This induces chemo-resistant dormancy in the sojourning cancer cells (38, 105, 113, 114) (Figure 1B). Dormancy occurs at two levels (101, 108): (a) at the single-cell level, in which the dormant cancer cells exist in a quiescent state of G0 – G1 arrest (101), with increased *MAPK p38* and decreased *ERK*, (conventional dormancy); and (b) at the population level, in which cancer cell proliferation is balanced by apoptosis (dynamic dormancy) resulting in an overall unchanged total cancer cell number (115), i.e., immune equilibrium (116, 117).

Our research found that thymic-sojourning disseminated solid tumor cells show a heterogeneous dormancy phenotype, some being quiescent with features of conventional dormancy, such as increased ratio of p38/ERK (activation of p38 and inhibition of ERK), inducing tumor growth arrest (113, 118, 119), while some either propagate or undergo apoptosis with features of dynamic dormancy (37). Together, chemotherapy-induced acute thymic involution provides a chemo-resistant microenvironment for tumor dormancy, creating a pre-metastatic reservoir. Although the distinct dormancy mechanism underlying the heterogeneity of dormant tumor cells (being quiescent and dynamic) needs further investigation, these observations provide a new therapeutic target for preventing cancer relapse and metastasis.

## Potential Therapeutic Strategies by Modifying Thymic Functions

Since cancer is derived from self-tissues, pathogenic tumor cells are oftentimes carrying “self”-antigens, i.e., TAAs, and can be recognized by most self-reactive Teff cells that are deleted by negative selection in the thymus. Thus, this has led several groups to posit that disruption of central tolerance might further the ability of the T cell compartment to combat cancers (87, 120–122). In this regard, most of the recent studies focus on targeting *Aire*-expressing mTECs in the thymus.

Medullary TECs highly express *Aire*, allowing them to promiscuously present self-antigens to self-reactive T clones during negative selection for central tolerance establishment (13, 21, 123). Though the full scope of this process remains to be elucidated, it is readily accepted that Aire deficiency facilitates increased self-reactive T cell release enhancing immunity to certain cancers. One recent technique targets mTECs specifically via anti-RANK-Ligand treatments, which transiently deplete *Aire*-expressing mTECs (22, 121, 124). Because the anti-RANK-Ligand reagent is already FDA-approved, albeit for osteoporosis (125), it has potential to be easily translated to cancer patients. This strategy is also promising because the depletion is brief, with mTECs normally replenished within 2 weeks (126, 127) and full recovery observed 10 weeks after cessation of anti-RANK-Ligand treatment (22). This tactic was tested in animal models of melanoma, since several of the melanoma antigens, including gp100 and TRP-1, are controlled by *Aire* (23, 122) and up-regulated in melanoma cells (122). Importantly, many of these studies used anti-RANK-Ligand in combination with peripheral therapies, such as checkpoint inhibitors, demonstrating greatly improved outcome in comparison to peripheral treatment alone. However, it is obvious that central therapy alone is not sufficient for tumor immunotherapy (121).

One caveat to this type of strategy is the recent finding that other transcriptional regulators are implicated in promiscuous self-antigen expression in the thymus, for example, forebrain embryonic zinc fingerlike protein 2 (Fezf2) (128). There are not many reports on what Fezf2 disruption would accomplish in regards to heightened TAA targeting as observed with the above Aire-targeting studies. There is evidence that Fezf2 is independent of the RANK/CD40/Aire axis which implies that an anti-RANK-Ligand therapy may not be as effective for disrupting Fezf2-dependent self-antigen expression (129).

The obvious risk for disruption of central tolerance is increased incidence of autoimmunity (130, 131), which is one of the underlying players in inflammaging in the elderly (66). This is clearly seen in patients who have mutations in *AIRE* (132) and has been recently demonstrated in mice who lack Fezf2 (128). Another challenge to strategies that manipulate central tolerance is that some TAAs are not under the control of *Aire*, such as TRP-2 (122), and some may be under the regulation of factors that have yet to be identified.

Additionally, we know that tumor antigens not only include TAAs (“self”-antigens), but also TSAs (“foreign”/neo-antigens), which are recognized by T cells as foreign antigens (133, 134). Therefore, deletion of *Aire* expression cannot induce antitumor immunity to non-*Aire*-controlled TAA-bearing tumors nor for TSA-bearing tumors. This limits the scope of cancers that would benefit from such a strategy, and also supports studies that use combinative central and peripheral immunotherapies. Finally, it is important to also take age-related peripheral changes into account, as many other age-related changes may offset the potential benefits of such central tolerance manipulation therapies. Therefore, several potential avenues of research remain for this type of cancer immunotherapy.

## Conclusion and Outstanding Questions

We have briefly reviewed some of the potential impacts of thymic involution (chronic age-related or acute chemotherapy-induced) on cancer and attempt to pave the way for further studies in tumor immunology. Since cancer and thymic atrophy are both associated with age, there is potential for a deeper connection. For instance, it is interesting to consider that most cancers develop in older adults, long after thymic involution has progressed. Since thymic involution is associated with declined mTEC cellularity and *Aire* expression in mTECs (66, 135), it raises the question of why there is not a natural increase in antitumor immunity in the elderly due to the defects in negative selection in the aged thymus. In addition, chemotherapy also induces TEC-impaired thymic involution (37) and declined *Aire* expression in tumor-bearing mice treated with doxorubicin (our unpublished observation). Why, then, do we not see enhanced antitumor T cell generation? Further, estrogen has recently been identified as a repressor of *Aire* (136, 137), possibly explaining the sex-related tendencies for higher autoimmune disease incidence in women. Does this correlate with a lower incidence for development of certain TAA-expressing cancers in post-menopausal women? In addition, whether we can manipulate thymic function to better target tumor-infiltrating Treg cells by weakening tTreg generation or harness newly generated Teff cells to home to the tumor is in need of further study. Finally, since the tumor microenvironment exerts such strong immunosuppressive signals, how can immunotherapies be tailored to overcome those signals in a tumor-specific manner without breaking peripheral tolerance completely. Moreover, many important questions remain in our understanding of the crosstalk of aging, cancer, and the impacts of thymic involution on late-life cancers.

## Author Contributions

D-MS: conceptualization and supervision. WW, RT, and D-MS: writing the original draft. OS: provide assistances. RT: visualization and proofreading.

## Conflict of Interest

The authors declare that the research was conducted in the absence of any commercial or financial relationships that could be construed as a potential conflict of interest.

## Abbreviations

AIRE: autoimmune regulator
ERK: extracellular signal-regulated kinases
MAPK: mitogen-activated protein kinases
MHC-II: major histocompatibility complex class-II
mTECs: medullary TECs
PD-1: programmed cell death protein 1
PD-L1: programmed death-ligand 1
pTreg: peripheral Treg cells
SA-T: senescence-associated T cells
TAA: tumor-associated antigen
Tcon: conventional T cells
TCR: T cell receptor
TECs: thymic epithelial cells
Teff: T effector cells
Treg: regulatory T cells
TSA: tumor-specific antigen
tTreg: thymic regulatory T cells.
